# Few geriatric heart failure patients investigated according to clinical guidelines: a retrospective review of patient records

**DOI:** 10.1186/s12877-023-03773-w

**Published:** 2023-03-21

**Authors:** Marianne Reimers Wessberg, Åke Seiger, Johan Fastbom, Maria Eriksdotter

**Affiliations:** 1 Department of Neurobiology, Care Sciences and Society, Division of Clinical geriatrics, Karolinska Institutet, Stockholm, Sweden; 2 Stiftelsen Stockholms Sjukhem, Mariebergsgatan 22, SE-112 19 Stockholm, Sweden; 3 Aging Research Center, Department of Neurobiology, Care Sciences and Society, Karolinska Institutet and Stockholm University, Stockholm, Sweden; 4 Theme Inflammation and Aging, Karolinska University Hospital, Huddinge, Sweden

**Keywords:** Heart failure, Echocardiography, Etiology, Preserved or reduced ejection fraction, Geriatric patients, Heart failure investigation, Collaboration between cardiologists and geriatricians

## Abstract

**Background:**

Research on heart failure (HF) has often focused on younger patients. The aim of this study was to analyze extent of investigation and treatment among older patients prior to referral to inpatient geriatric care for worsening of HF.

**Methods:**

Data on etiology, ejection fraction (EF) by echocardiography (ECHO), level of functioning according to New York Heart Association (NYHA), analysis of N-terminal-pro-brain natriuretic peptide (NT-Pro-BNP), ongoing treatment, adherence to guidelines, and information from previous caregiver were collected from patient records prior to admission from a sample of 134 patients.

**Results:**

Few patients had been examined by a cardiologist (14%) during the year prior to referral. EF assessment had been performed in 78% (*n* = 105). The patients were categorized as having HF with reduced (HFrEF 28%), preserved (HFpEF 53%) or mid-range (HFmrEF 19%) EF. HFpEF patients had older EF assessments (mean 517 days) than those with HFrEF (385 days). In 61% (*n* = 82) at least one assessment with NT-Pro-BNP had been performed, being older among patients with HFpEF (290 days vs 16 days).

There was a strong positive correlation (OR 4.9, *p* = 0.001) between having recent assessments of EF and NT-Pro-BNP (*n* = 30, 21%) and being presented with etiology in the referral, adjusted for EF, age, sex, and comorbidity.

Among the HFrEF patients, 78% were treated with ACEI/ARB and BB according to ESC guidelines but reaching only half of target doses. In the HFpEF group the corresponding treatment was 46%. Among patients with EF ≤ 35% only 14% were treated with mineral receptor antagonists, ie low adherence to guidelines.

**Conclusions:**

HF care in this population of older individuals showed deficiencies. There was little contact with cardiologists, lack of information of etiology in referrals and low adherence to treatment guidelines. Improving adherence to HF guidelines regarding investigation and treatment for HF in older people is therefore urgent and calls for more collaboration between specialists in cardiology and geriatric medicine.

## Background

Heart failure (HF) is globally the most common cause for admission to hospital for patients 65 years and older in high income countries [1]. There are two main types of heart failure, HF with reduced ejection fraction (EF < 40%; HFrEF) and HF with preserved ejection fraction (EF ≥ 50%; HFpEF). As the prevalence of HF increases with age, the proportion of patients with HFpEF is increased in relation to the proportion of patients with HFrEF [2]. Over 50% of all HF [3] and over 70% of all HF patients older than 65 years have been reported to belong to the HFpEF group [3, 4]. This group is more often female and has more often a history of hypertension and atrial fibrillation [5, 6]. Yet, it has been reported that geriatric patients are under-represented in major HF clinical trials with an average age of enrollment in HF randomized clinical trials almost 20 years younger than the average age in epidemiological cohorts [7].

The two cornerstones in diagnosing HF are assessment of EF and NT-pro-BNP, according to recommendations from the European Society of Cardiology (ESC). Assessment of functioning level according to the New York Heart Association (NYHA) is further required to determine the level of HF and choice of treatment. N-terminal-pro-brain natriuretic peptide (NT-pro-BNP) can be used to follow the effect of treatment, but also to predict worsening [7]. However, compliance to these guidelines varies. Rutten et al [8] reported that general practitioners performed fewer examinations of these patients, compared to cardiologists, and also showed that HF patients in primary care were older and often female. Stork et al [9] showed that in ambulatory settings in Germany only 15% of older HF patients were diagnosed by a cardiologist. Several studies have reported that older patients with HF may be under-diagnosed [10, 11]. Smeets et al [12] reported that in primary care, analyses of NT-pro-BNP and echocardiography (ECHO) in older patients were underutilized, leading to both under- and over-diagnosing. Munoz and colleagues investigated HF patients in primary care and found that only 8.5% had their EF noted in the medical records [13]. The lack of documentation on EF was associated with a higher risk of adverse outcomes. These patients tended to be older, socio-economically disadvantaged and more frail [13].

The aim of the present study was to investigate from patient records in a cohort of older patients hospitalized for worsening of their HF and referred to geriatric inpatient care, whether the type of HF was known, what diagnostic investigations had been performed and what treatment of HF was prescribed prior to admission to geriatric care, and finally if this information was available in the referral to geriatric care**.**

## Material and methods

### Subjects and setting

Data on HF diagnostics and treatment (see below) were collected from a consecutive sample of patients with HF prior to their referral to geriatric inpatient care for treatment of worsening of their previously diagnosed HF. Patients referred to one geriatric clinic in the Stockholm region, Sweden, during the period from 1st of July 2015 to 30th of June 2016 were included if they met the inclusion criterion of being referred due to a main diagnosis of HF (codes I50.0, I50.1, I50.9 and I11.0, according to the International Statistical Classification of Diseases and related Health Problems, tenth revision, Clinical Modification (ICD-10-CM) [14].

If patients were referred to inpatient geriatric care more than once during the study period, only data prior to the first geriatric inpatient care episode for HF was collected. During the study period, 280 patients with HF as the main diagnosis were referred to inpatient geriatric care. All patients received a number between 1 and 280. Using a random number table, constructed via the webpage www.slump.nu, 135 patients were selected. One of the selected patients was excluded due to erroneous registration. Thus, data from 134 patients were collected.

### Data collection

Data were extracted from the EMR system TakeCare (CompuGroup Medical, Stockholm, Sweden), which is in use in most inpatient hospital clinics and primary care centers in Stockholm, or from Cosmic (Cambio Healthcare Systems, Stockholm, Sweden), which is the EMR used by the main provider of data on HF of the included patients.

The following data from patient charts, from any hospital or primary care center in the Stockholm region prior to referral to inpatient geriatric care for HF worsening, were registered: age, sex, referral origin, number of inpatient care episodes during the last 12 months, days since last EF assessment by echocardiography (ECHO), level of EF, number of days since last analysis of NT-Pro-BNP, classification according to NYHA, recent (less than a year) contact with a cardiologist, comorbidity index according to Charlson [15], and also the presence of atrial fibrillation, myocardial infarction, hypertension or diabetes mellitus.

In addition, levels of NT-pro-BNP, hemoglobin and creatinine prior to the care episode were registered.

HF-related pharmacological treatment at the time of referral was also collected, i.e., angiotensin converting enzyme inhibitors (ACEI: Enalapril, C09AA02, Ramipril C09AA05), angiontensin-2-blockers (ARB: Cozaar C09CA01, Candesartan C09CA06, Irbesartan C09CA04), beta blockers (BB: Bisoprolol C07AB07, Metoprolol C07AB02, Atenolol CA7AB03) and mineralocorticoid receptor antagonists (MRA: Spironolactone C03DA01). Data on treatment with diuretics (Furosemide C03CA01, Hydrochlorothiazide C03AA03, Bendroflumethiazide + potassium chloride C03AA01, Amiloride + Hydrochlorothiazide C03EA01) was collected when available (86%) 14 days prior to admission.

Types of HF were defined according to the criteria by ESC [16]: Patients with an EF < 40% were defined as HFrEF, patients with 40% ≥ EF < 50% as HFmrEF and patients with EF ≥ 50% as HFpEF.

### Statistical analyses

Descriptive analyses concerning demography and investigations were performed. Tests used were pr-test (chi2) for calculation of differences in proportions between two groups, Student’s t-test for calculation of means between two groups and logistic regression’s test used for calculation of the odd’s ratio between recent assessments and referral information. Comparisons were made between patients with Student’s t-tests comparing HFrEF to non HFrEF and HFpEF to non HFpEF. Analyses were performed using Stata (College Station, Texas, USA) version 10. A *p*-value < 0.05 was considered statistically significant.

## Results

### Patient population

Information on HF diagnostics and treatment prior to referral was collected from 134 patients referred with HF as the main diagnosis to an inpatient geriatric clinic. The mean age was 85 years (SD 8.0), and the majority was female (Table 1). About three quarters of the patients were referred from emergency units or internal medicine clinics, 15% from primary care, and about 10% directly from home through itinerant primary care. The average number of inpatient care episodes for HF during the last 12 months prior to the present referral was 3.8 (range 0 to 29).

**Table 1:** Characteristics of the study population (*n* = 134)

General features	Patients referred to a geriatric ward with HF (***n*** = 134)
Females, n (%)	75 (56)
Age, years, mean (SD)	85.0 (8.0)
Referral from hospital, n (%)	100 (75)
Referral from primary care, n (%)	20 (15)
Directly from home, n (%)	14 (10)
Contact with cardiologist during the last year, n (%)	19 (14)
Care episodes during the last 12 months, mean (SD)	3.8 (4.4)
Charlson comorbidity index score, mean (SD)	3.7 (3.2)
Atrial fibrillation, n (%)	93 (69)
Myocardial infarction, n (%)	36 (27)
Hypertension, n (%)	54 (40)
Diabetes Mellitus, n (%)	39 (29)
**Information in referral to geriatric inpatient care**	
Etiology, n (%)	37 (27.6)
EF, n (%)	21 (15.7)
NT-pro-BNP, n (%)	14 (10.4)
**Investigations prior to admission to a geriatric ward**	
Ejection fraction, assessed anytime, n (%))	105 (78)
Ejection fraction, days since assessment, mean (SD)	463 (745)
NT-pro BNP, assessed anytime, n (%)	81 (60)
NT-Pro-BNP, days since assessment, mean (SD)	156 (460)
Recent (3 months or shorter) investigation, n (%) with assessment of EF and NT-Pro-BNP	30 (22)
NYHA assessment ever performed, n (%)	4 (3)
Never performed EF assessment nor NT-pro-BNP, n (%)	11 (8)
Contact with cardiologist about HF within 12 months, n (%)	19 (14)
**Laboratory analyses at admission to geriatric ward**	
Hemoglobin mg/L, mean (SD)	115.4 (18.9)
Creatinine, μmol/L, mean (SD)	104.8 (55.9)
eGFR, mL/min/1,73 m^2^, mean (SD)	64.7 (25.7)
**Ongoing HF treatment at admission to geriatric ward**	
ACEI or ARB, n (%)	86 (64)
BB, n (%)	107 (80)
MRA, n (%)	24 (18)
Diuretics, n (%)^a^	118 (86.5)

^a^Recorded 14 days before admission

The Charlson comorbidity index showed a mean of 3.7. In addition to HF, atrial fibrillation was the most common cardiovascular comorbidity (69%), followed by hypertension (40%), and myocardial infarction (27%). The prevalence of diabetes mellitus was 29%.

During the year prior to referral, a cardiologist had been consulted about the HF in 14% of the patients.

### Investigations performed

Data on investigations (ECHO, NT-pro-BNP, NYHA) were traced in the records. In the case of ECHO only those with information of EF was included. The oldest information on assessments were from 1996 (ECHO), 2011 (NT-pro-BNP) and 2013 (NYHA).

Prior to referral to inpatient geriatric care, 105 patients (78%) had had an ECHO performed (with a mean of 463 days prior to referral), 81 patients (60%) had had a NT-Pro-BNP analysis performed (with a mean of 156 days prior to referral, Table 1). Only 4 patients (3%) had had a NYHA assessment performed.

**Fig. 1:**
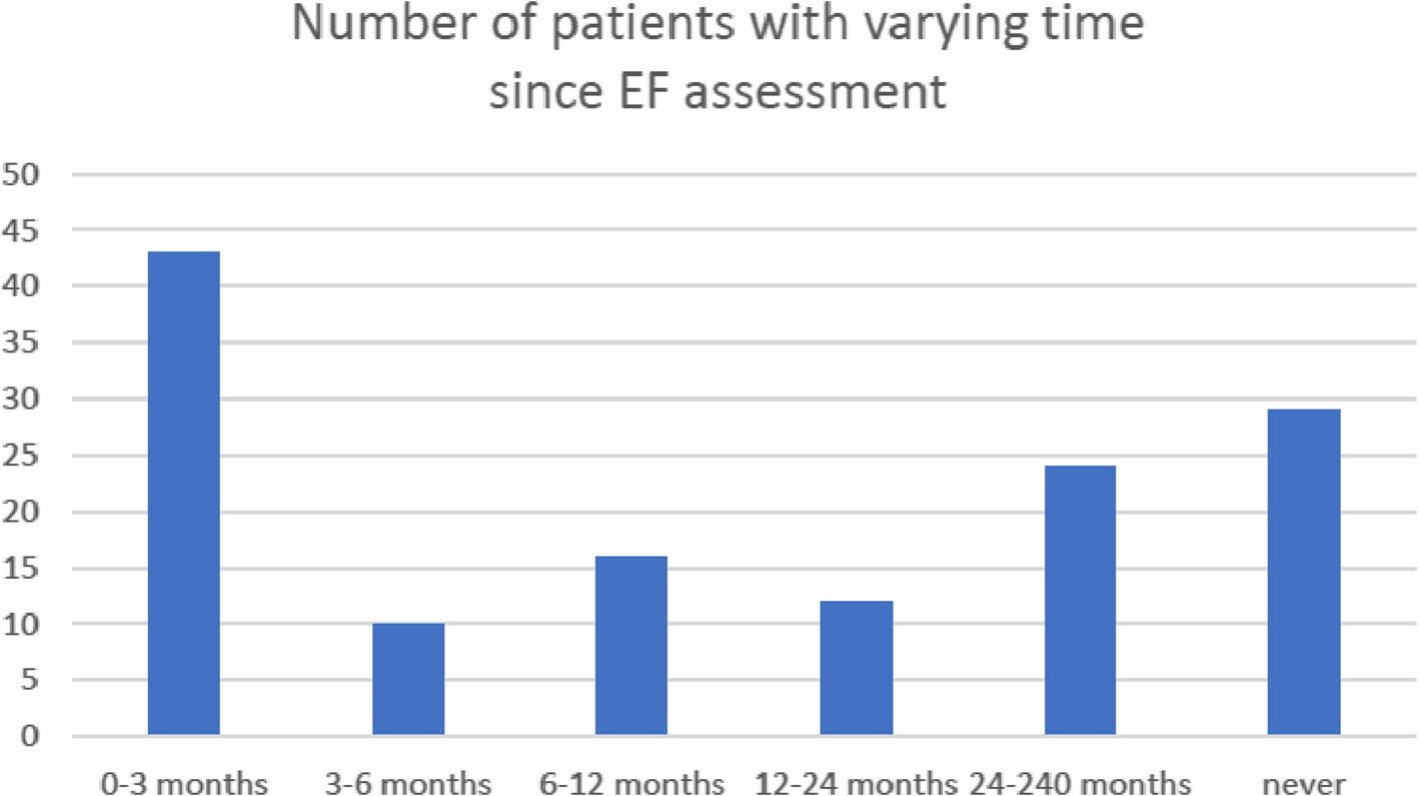
Shows the number of patients with an EF assessment performed prior to admission to geriatric care and the age of the assessment. The number of patients with no EF assessment at all (*n* = 29) is also shown

**Fig. 2:**
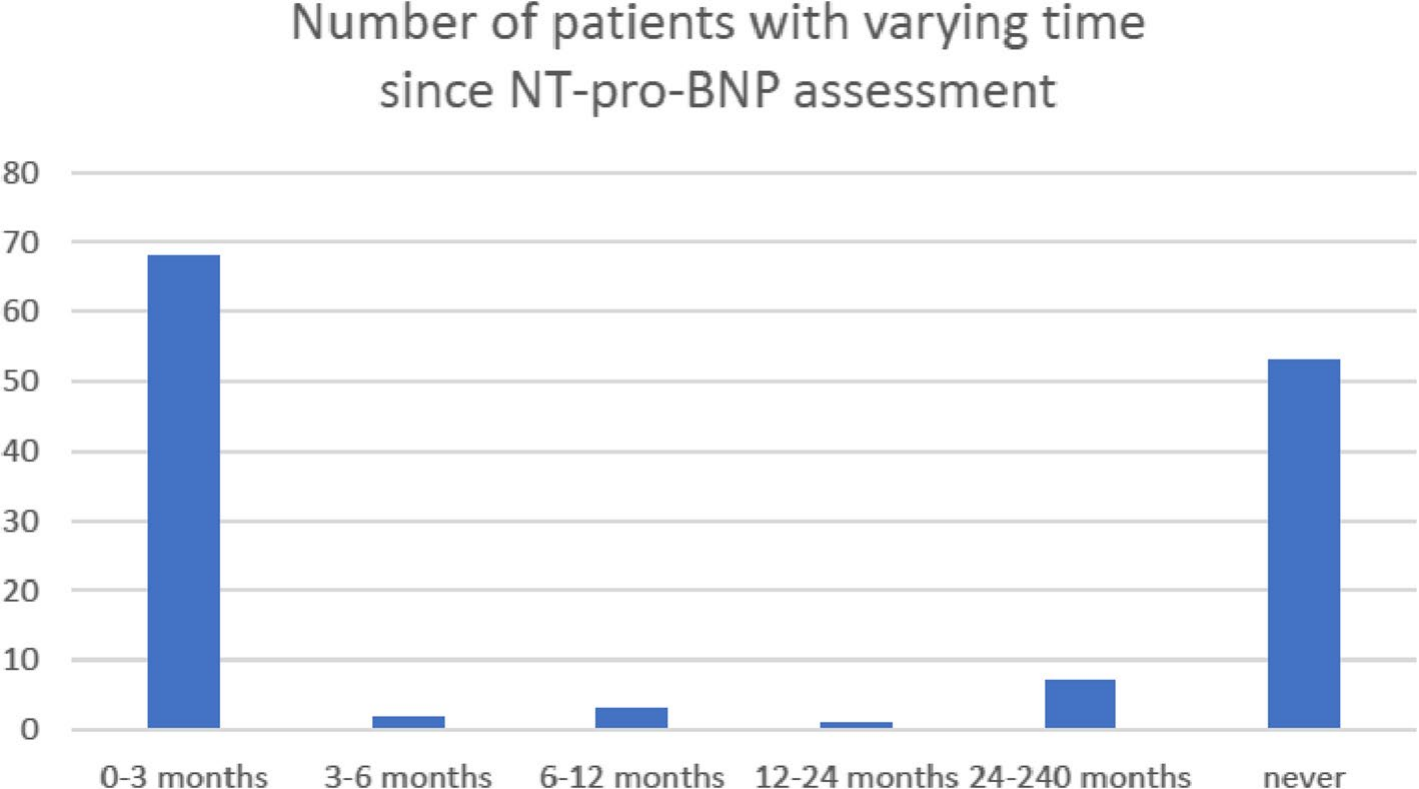
Shows the number of patients with analysis of NT-pro-BNP prior to admission to geriatric care. The majority had an assessment performed within 3 months prior to admission but 53 had never had an analysis performed

In 32% (*n* = 43) of the patients, ECHO was performed 3 months or less prior to referral. In 15% (*n* = 20) of the patients the ECHO examinations were 2 years or older and in 22% (*n* = 29) an ECHO had never been performed (Fig. 1). Figure 2 shows that 51% (*n* = 68) of the patients had had an analysis of NT-pro-BNP performed within 3 months, but in 39% (*n* = 52) there were no results on NT-pro-BNP in the records.

Considering the variation of age of the ECHO and NT-pro-BNP assessments shown in Figs. 1 and 2, a recent investigation was defined as assessment with ECHO and NT-pro-BNP within 3 months.

Eight percent of the patients were neither assessed with ECHO nor with NT-pro-BNP. This group differed significantly from the other studied patients by having fewer care episodes during the last year (1.2 vs 4.2), no previous myocardial infarction (0% vs 28.4%) and no contact with a cardiologist during the last year (0% vs 17%).

A subgroup of patients (*n* = 30, 22%) had had both an ECHO investigation and an NT-pro-BNP analysis performed within 3 months of the referral, i.e., had undergone a recent investigation (Table 2).

**Table 2:** Comparison of investigations made prior to the geriatric care episode between groups with different EF

Investigations	EF < 40% (***n*** = 29)	≥40%EF < 50% (***n*** = 20)	EF ≥ 50% (***n*** = 56)	EF unknown (***n*** = 29)
NT-pro-BNP, ever, n (%)	15 (52)	12 (60)	39 (69)	15 (52)
NYHA ever, n (%)	2 (0.7)	0 (0)	2 (3.6)	0
Recent investigation^a^, n (%)	10 (34.5)	6 (30)	14 (25)	0
Complete investigation^b^, n (%)	1 (0.3)	0 (0)	0 (0)	0

^a^EF assessment and NT-pro-BNP analyses within the last 90 days prior to referral

^b^EF assessment, NT-Pro-BNP and NYHA assessment ever performed

### Comparisons between patients according to EF

A hundred and five patients (78%) had had their EF assessed with ECHO at any time prior to admission. Based on the ECHO examination, 29 patients (28%) were categorized as HFrEF (EF < 40%), 20 patients (19%) as HFmrEF (40% ≥ EF < 50%) and 56 patients (53%) as HFpEF (EF ≥ 50%). Characteristics and comparisons between the groups are presented in Table 3.

**Table 3:** Comparisons of demographics, clinical features and investigations of groups defined according to type of HF: HFrEF, HFmrEF, HFpEF regarding variables at referral. *P*-values for comparisons between HFrEF vs non-HFrEF and HFpEF vs non-HFpEF are shown

General features	HFrEF (***n*** = 29)	HFmrEF (***n*** = 20)	HFpEF (***n*** = 56)	p/z-value HFrEF-non HFrEF	p/z-value HFpEF-non HFpEF
Proportion of females, n (%)	15 (51.7)	13 (65.0)	32 (57.1)	0.488	1.000
Age, years, mean (SD)	83.7 (8.7)	83.1 (7.2)	86.3 (6.7)	0.286	0.051
Care episodes during the last 12 months, mean (SD)	3.6 (3.6)	5.3 (5.6)	4.2 (4.8)	0.383	0.936
Referral from hospital, n (%)	22 (75.8)	13 (65.0)	42 (75.0)	0.721	0.683
Referral from primary care, n (%)	5 (17.2)	6 (30.0)	5 (10.7)	0.858	0.113
Directly from home, n (%)	4 (13.8)	1 (5.0)	9 (16.1)	0.372	0.112
Contact with cardiologist regarding HF < 12 months, n (%)	7 (24.1)	4 (20.0)	8 (14.3)	0.325	0.283
**Comorbidities**					
Charlson comorbidity index, mean	4.24	3	4.01	0.342	0.623
Atrial fibrillation, n (%)	51 (51.7)	20 (100)	48 (66.1)	0.002**	0.600
Hypertension, n (%)	8 (27.6)	8 (40.0)	29 (50)	0.117	0.074
Myocardial infarction, n (%)	12 (41.4)	5 (25.0)	14 (25)	0.047*	0.282
Diabetes mellitus, n (%)	20 (27.6)	4 (20.0)	19 (33.9)	0.840	0.295
**Investigations prior to admission**					
Ejection fraction, time since assessment, days mean (SD)	385 (582)	454 (701)	517 (844)	0.484	0.477
Ejection fraction, %, mean (SD)	26.6 (7.4)	42.6 (2.9)	53.4 (3.2)	0.000*	0.000*
NT-Pro BNP any time, n (%)	15 (51.7)	12 (60.0)	39 (69.4)	0.240	0.275
NT-pro BNP, time since assessment, days, mean (SD)	16 (20)	16 (25)	290 (627)	0.011*	0.011*
Recent (within 3 months) investigation of ECHO and NT-pro-BNP, n (%)	10 (34.5)	6 (30.0)	12 (21.4)	0.268	0.029*
**Information on HF in referral**					
Etiology, n (%)	16 (55.2)	4 (20.0)	15 (26.7)	0.003**	0.131
Ejection fraction, n (%)	11 (37.9)	4 (20.0)	6 (10.7)	0.004**	0.013*
NT-Pro-BNP, n (%)	3 (10.3)	4 (20.0)	3 (5.4)	0.861	0.134
**Laboratory analyses at admission**					
NT-Pro-BNP, ng/L, mean (SD)	12,283 (7464)	7930 (8082)	4422 (5650)	< 0.001***	< 0.001***
Hemoglobin mg/L, mean (SD)	118 (22)	114 (11)	113 (16)	0.310	0.347
Creatinine, μmol/L, mean (SD)	93 (27)	111 (56)	116 (73)	0.112	0.193
eGFR, mL/min/1,73 m^2^, mean (SD)	72 (28)	57 (23)	59 (23)	0.014*	0.144
**Ongoing pharmacological treatment at referral**					
ACEI/ARB, n (%)	24 (82.8)	14 (70.0)	35 (62.5)	0.018*	< 0.001***
BB, n (%)	27 (93.1)	20 (100)	39 (69.6)	0.025*	< 0.001***
MRA, n (%)	5 (17.2)	2 (10)	14 (25)	0.666	0.174
ACEI/ARB and BB, n (%)	23 (79.0)	14 (70.0)	26 (46.4)	0.003*	0.003*
ACEI/ARB, BB and MRA, n (%)	4 (13.8) (For HFrEF< 35%)	1 (5.0)	9 (16.1)	0.510	0.510
Diuretics, n (%)^a^	25 (86.2)	17 (85)	49 (87.5)	0.934	0.791
**Doses related to target doses**					
ACEI/ARB, % (SD)	52.8 (31)	46.1 (31)	54.8 (32	0.994	0.510
BB, % (SD)	53.2 (32)	66.3 (30)	51.7 (45)	0.786	0.289

^***^p-value < 0.001

^**^p-value 0.001–0.009

^*^p-value 0.010–0.049

^a^Measured 14 days before admission

There were no significant differences in total comorbidity according to the Charlson index between HFrEF, HFmrEF and HFpEF. However, analyzing specific comorbidities between the two main groups HFrEF and HFpEF, we found that HFrEF more often had suffered a myocardial infarction (41% vs 25%), while HFpEF more often had hypertension (50% vs 28%) and atrial fibrillation (66% vs 52%).

HFpEF patients had significantly less often a recent investigation of ECHO or NT-pro-BNP compared with the non-HFpEF group (*p* < 0.029). There was a significant difference in average age of the analyses of NT-pro-BNP, with HFpEF counting 290 days, in contrast to 16 days prior to admission for the other groups (Table 3). Very few patients (*n* = 4) had information about NYHA in the records.

### Information in referral

HFrEF patients were significantly more often presented with HF etiology (55% vs 25%) and EF (38% vs 13%) in the referrals compared to the non-HFrEF group (Table 3).

A separate analysis showed a strong positive correlation (OR 4.9, *p* < 0.001) between having a recent investigation of EF and NT-pro-BNP within 3 months prior to referral and being presented with etiology in the referral to inpatient geriatric care, adjusted for level of EF, age, sex, and comorbidity.

### Laboratory analyses

Prior to referral, HFrEF patients were presented with significantly higher levels of NT-pro-BNP (mean 12,283 vs 5264 ng/L, *p* = 0.001), but with significantly better e-GFR (72 vs 58 ml/min/1,73m^2^, *p* = 0.014) compared to non-HFrEF (Table 3).

Pearson’s correlation test showed that age and NT-pro-BNP among the patients in this study were independent (r = 0.111).

### Pharmacological treatment and adherence to guidelines

HFrEF patients were more often treated with ACEI or ARB compared with the non-HFrEF group (83% vs 64%). This was also true of treatment with betablockers (93% vs 77%, Table 3).

To assess the degree of adherence to guidelines for HFrEF, data on the NYHA classification is needed. Our data here shows that NYHA is rarely recorded.

Although the NYHA assessments were rarely performed, these patients had HF symptoms since they were admitted to a geriatric ward for further HF treatment. Thus, it can be assumed they belong to NYHA groups II-IV. Therefore, all HFrEF patients in the study should, according to guidelines, be treated with ACEI/ARB and BB. This was the case for 79% of this subgroup, see Table 3. Furthermore, HFrEF patients with EF ≤ 35% should be treated with ACEI/ARB, BB and MRA according to guidelines. This was the case among 14% of the patients, (Table 3).

When analyzing the doses of these drugs patients in the three groups were in average treated with a little over 50% of the target doses, see Table 3, with the exception of the HFmrEF group which was treated with 46% of the ACEI/ARB target doses, but 66% of the target doses of BB.

The guidelines for HFmrEF and HFpEF are less clear and conditioned to comorbidities such as atrial fibrillation, diabetes, and chronic obstructive pulmonary disease. Data on the HFmrEF and HFpEF groups here was not sufficient to evaluate adherence to guidelines.

## Discussion

Here we have presented five major findings prior to admission of patients referred to inpatient geriatric care for worsening of HF:

i) Only a minority (14%) of older HF patients referred to inpatient geriatric care had been seen by a cardiologist during the year prior to referral
ii) Very few (3%) had had a NYHA classification recorded prior to admission.
iii) A majority of the patients was investigated with EF assessment (78%) and NT-pro-BNP (60%), but these investigations were not recent, with a mean of 463 and 156 days prior to admission respectively, especially among persons with HFpEF.
iv) A strong positive correlation was found between having an assessment of EF and NT-pro-BNP within 3 months – which occurred in 22% of the cases – and being presented with etiology in the referral.
v) Assuming that all HFrEF patients belonged to NYHA class II-IV we found that 79% were treated with ACEI/ARB and BB, according to guidelines, but only to a little more than 50% of target doses. Patients with EF ≤ 35% were treated according to guidelines in 14% of the cases.

### The patient population

The features of HF patients in our study resembled those of general geriatric patients in Sweden concerning age, sex, and referral origin [17] as previously reported recently.

In general, the risk of readmission within 30 days among patients in medical care has been reported to be 20% [18]. Among HF patients the risk has been shown to be even higher, with an incidence of nearly 25% [19]. Our finding with 3.8 care episodes during the year prior to referral suggest that our cohort with geriatric HF patients are high consumers of inpatient care.

The finding that HFrEF had more often myocardial infarction in their history compared to HFpEF is in line with other studies as is our finding that HFpEF more often had hypertension and atrial fibrillation compared to HFrEF [20].

### Investigations

Only a small minority (14%) of the patients had had contact with a cardiologist about their HF during the last 12 months prior to referral, which is in line with the findings by Stork et al [9] who reported that 14.8% of HF diagnoses in Germany were made by cardiologists. This lack of contact is remarkable since the regional care chain for HF in Stockholm presumes a cardiologist’s assessment as a principal rule.

In this study 78% of the patients had had an EF assessment within an average time period of 463 days prior to referral. We have not found any recommendations on periodicity of EF assessments. However, the average age of the EF assessments seems high, considering that the referrals were due to acute deterioration for patients with frequent inpatient care episodes.

While ECHO assessment is costly and not always accessible, NT-pro-BNP is available and cheap. Since HFpEF is more difficult to diagnose with ECHO than HFrEF [4], there is more reason to evaluate these patients with NT-pro-BNP. The present results, however, showed the opposite, with an average of 290 days since the last NT-pro-BNP assessment among HFpEF patients compared to 16 days among the non-HFpEF group. Similar results have according to our knowledge not been presented in other studies.

There are several possible explanations for the seemingly poorer investigations of HFpEF. Older patients with preserved EF may be overlooked, presenting with traditionally less typical HF symptoms. The reasons for renewing ECHO assessments may be less obvious if EF is preserved, although a first assessment should be done to characterize the HF. However, the reason for the low use of NT-pro-BNP assessments in this group is unclear. According to Islam et al [21], NT-pro-BNP is clearly correlated to the severity of HFpEF. One may speculate that, since the etiology of HFpEF typically is more complex, less attention may be paid to this patient group and the frequent features of high age and multiple comorbidities may add to this.

We found a subgroup of HF patients being investigated with ECHO and NT-Pro-BNP within 90 days prior to admission to geriatric care. In a separate analysis we found a significant positive correlation between such a recent investigation and the presentation of etiology in the referral. Our interpretation is that a recent investigation increases the etiological thinking and ambition for the treatment of patients, regardless of age, sex, EF, and comorbidity, and primes for a better understanding and thereby a more adequate treatment of the patients. The correlation may be regarded as self-evident, nevertheless the risks of losing data in transition between caregivers are well known. In an up-coming study, we plan to follow the content in the referrals to geriatric care and the transition of information back to primary care.

Eight percent of the patients had neither been investigated with ECHO nor NT-Pro-BNP. The foundation for the HF diagnosis was, however, weak in these patients and they may have been misdiagnosed.

The almost non-existent use of NYHA assessments is remarkable. A reason for this could be that elderly patients may have several causes for limitations in strength and fitness, which may make the NYHA classification seem less useful for evaluating the level of HF. This could affect geriatric patients more than other patient groups. Another explanation could be that symptoms in older people are more vague, which may make the health staff less alert to the long-term serious nature of the condition and thus the need for NYHA assessment.

### Treatment

A majority of HFrEF patients was treated with ACEI or ARB and BB, but in average to little more than half of the target doses according to the ESC guidelines for treatment of HFrEF. Possible reasons for the low doses may be frailty, low blood pressure, high comorbidity, impaired kidney function and high age, but also short care episodes. The lack of updated current data on the HF status with ECHO and/or NT-Pro-BNP, as well as lack of contact with a cardiologist may have impeded the intentions to titrate the doses.

Also, a large share (46%) of HFpEF patients was treated with ACEI or ARB and BB. It is likely that these patients were treated with ACEI or ARB mainly due to hypertension or diabetes with microalbuminuria and with BB due to atrial fibrillation.

Among HFmrEF patients we found that they were treated with 66% of target doses of BB. This could correlate to the fact that all patients in this group had atrial fibrillation and therefore a need of pulse control.

Assuming that all patients in our study would, if assessed, have been classified as NYHA II-IV, we can conclude that 79% of the patients with HFrEF were treated with both ARB/ACEI and BB, as recommended by ESC, although not in recommended doses.

We can also, in the present study, conclude that 86% of patients with EF ≤ 35% were not treated according to guidelines, since the ESC recommendation is to treat such patients with MRA, and this was only the case for 14%. This finding is in line with those of Savarese et al [22], who showed that MRA was underused among HF patients. Factors associated with the under-use were, among others, low glomerular filtration rate, older age, lower income, and male sex.

Our study confirms the observations by Butrous and Hummel [23] and Abete et al [24], that geriatric HF patients are poorly investigated and treated compared to HF patients included in cardiological studies. This emphasizes the importance to identify the etiology of HF also for older patients with HF to improve treatment. In fact, since old people with HF often are high consumers of inpatient care, higher ambitions seem urgent.

### Study limitations

Even though careful collection and scrutiny of medical records were done, information may have been missed or overlooked.

There may also be relevant information about patients and decisions made, which are not presented in the patient records nor considered during the care episodes. Patients may also have sought care at private health centers with other documentation systems not available in this study.

The varying age and quality of the EF assessments could have influenced the distribution between HFrEF, HFmrEF and HFpEF at the time of our investigation, since HFrEF patients may receive more medical attention and therefore have a more reliable categorizing while the symptomatology of HFpEF is more vague and the need for EF assessment may be overlooked. Although a power analysis was performed prior to the study, the number of patients especially in the subgroup analyses was small, making the conclusions less robust.

The guidelines for HFmrEF and HFpEF are less clear and conditioned to comorbidities such as atrial fibrillation, diabetes, and chronic obstructive pulmonary disease. The collected data on the HFmrEF and HFpEF groups was not sufficient to evaluate adherence to guidelines.

Finally, we focused on geriatric patients with HF as a main diagnosis. Since the HF diagnosis commonly is contributory, there may be features of the contributory HF diagnosis and its treatment and care which were not included.

## Conclusions

Since HF is a heterogenous disease with varying etiology, comorbidities, prognoses and treatment guidelines, it is of vital importance that HF patients receive a relevant investigation to determine the etiology as a basis for further planning. In our study we found that most HF patients at referral to geriatric inpatient care lacked relevant information about their HF diagnosis important for their treatment and care.

It is unlikely that improved investigation and, hence, improved basis for treatment strategies can, at least in a shorter perspective, be managed only by cardiologists, due to the sheer volume of geriatric HF patients. Moreover, frailty and multimorbidity, commonly found in geriatric HF patients may also call for expertise in geriatric medicine. It is therefore desirable that investigation and treatment of HF in geriatric patients to a large extent will continue to be performed in primary and geriatric care. The aim must therefore be to increase adherence to HF guidelines both regarding assessment of HF status to obtain knowledge of the HF etiology and guidelines for treatment, although adjusted for geriatric patients, and to implement and support these ambitions in geriatric and primary care.

It is important to further study diagnostics, treatment, and care of older HF patients in inpatient geriatric care. Our next aim is therefore to investigate whether the geriatric care episodes do contribute to such well needed improvement in investigation and treatment.

## Data Availability

The datasets used and/or analysed during the current study available from the corresponding author on reasonable request.

## Abbreviations

HF: Heart failure
EF: Ejection fraction
ECHO: Echocardiography
HFpEF: Heart failure with preserved ejection fraction
HFrEF: Heart failure with reduced ejection fraction
HFmrEF: Heart failure with mid-range ejection fraction
NYHA: New York Heart Association, classification of functioning level among HF patients
NT-pro-BNP: N-terminal-pro-brain natriuretic peptide
ACEI: Angiotensin converting enzyme inhibitor
ARB: Angiotensin 2 receptor blocker
BB: Beta blockers
MRA: Mineral corticoid receptor antagonist
